# Co-Culture of Glomerular Endothelial Cells and Podocytes in a Custom-Designed Glomerulus-on-a-Chip Model Improves the Filtration Barrier Integrity and Affects the Glomerular Cell Phenotype

**DOI:** 10.3390/bios13030339

**Published:** 2023-03-03

**Authors:** Daan C. ‘t Hart, Dilemin Yildiz, Valentina Palacio-Castañeda, Lanhui Li, Burcu Gumuscu, Roland Brock, Wouter P. R. Verdurmen, Johan van der Vlag, Tom Nijenhuis

**Affiliations:** 1 Department of Nephrology, Research Institute for Medical Innovations, Radboud University Medical Center, 6500 HB Nijmegen, The Netherlands; 2 Department of Medical Biosciences, Research Institute for Medical Innovations, Radboud University Medical Center, 6500 HB Nijmegen, The Netherlands; 3 Biosensors and Devices Laboratory, Biomedical Engineering Department, Institute for Complex Molecular Systems, Eindhoven Artificial Intelligence Systems Institute, Eindhoven University of Technology, 5600 MB Eindhoven, The Netherlands; 4 Department of Medical Biochemistry, College of Medicine and Medical Sciences, Arabian Gulf University, Manama 329, Bahrain

**Keywords:** organ-on-a-chip, glomerulus, glomerulus-on-a-chip, co-culture, glomerular filtration barrier, crosstalk, biological barrier, glomerular endothelial cells, podocytes

## Abstract

Crosstalk between glomerular endothelial cells and glomerular epithelial cells (podocytes) is increasingly becoming apparent as a crucial mechanism to maintain the integrity of the glomerular filtration barrier. However, *in vitro* studies directly investigating the effect of this crosstalk on the glomerular filtration barrier are scarce because of the lack of suitable experimental models. Therefore, we developed a custom-made glomerulus-on-a-chip model recapitulating the glomerular filtration barrier, in which we investigated the effects of co-culture of glomerular endothelial cells and podocytes on filtration barrier function and the phenotype of these respective cell types. The custom-made glomerulus-on-a-chip model was designed using soft lithography. The chip consisted of two parallel microfluidic channels separated by a semi-permeable polycarbonate membrane. The glycocalyx was visualized by wheat germ agglutinin staining and the barrier integrity of the glomerulus-on-a-chip model was determined by measuring the transport rate of fluorescently labelled dextran from the top to the bottom channel. The effect of crosstalk on the transcriptome of glomerular endothelial cells and podocytes was investigated via RNA-sequencing. Glomerular endothelial cells and podocytes were successfully cultured on opposite sides of the membrane in our glomerulus-on-a-chip model using a polydopamine and collagen A double coating. Barrier integrity of the chip model was significantly improved when glomerular endothelial cells were co-cultured with podocytes compared to monocultures of either glomerular endothelial cells or podocytes. Co-culture enlarged the surface area of podocyte foot processes and increased the thickness of the glycocalyx. RNA-sequencing analysis revealed the regulation of cellular pathways involved in cellular differentiation and cellular adhesion as a result of the interaction between glomerular endothelial cells and podocytes. We present a novel custom-made glomerulus-on-a-chip co-culture model and demonstrated for the first time using a glomerulus-on-a-chip model that co-culture affects the morphology and transcriptional phenotype of glomerular endothelial cells and podocytes. Moreover, we showed that co-culture improves barrier function as a relevant functional readout for clinical translation. This model can be used in future studies to investigate specific glomerular paracrine pathways and unravel the role of glomerular crosstalk in glomerular (patho) physiology.

## 1. Introduction

The glomerulus is crucial for normal renal function by filtering the blood across the glomerular filtration barrier (GFB). The GFB comprises two cell types: fenestrated glomerular endothelial cells (GEnC) and visceral epithelial cells (podocytes). These two cell types are separated by the glomerular basement membrane, which mainly consists of collagen IV and laminin [1]. GEnC are covered by glycocalyx, a thick, negatively charged layer of carbohydrates. The glycocalyx plays an important role in glomerular function, for example by preserving glomerular structural integrity and affecting charge-selective glomerular permselectivity [2,3,4]. GFB injury is a crucial aspect of the pathogenesis of both acquired and hereditary forms of renal diseases, such as diabetic nephropathy and focal segmental glomerulosclerosis (FSGS) [5]. The aforementioned glomerulopathies are characterized by GEnC and podocyte injury, podocyte foot process effacement- and depletion, proteinuria, reduced glomerular filtration and eventually loss of kidney function [6,7,8]. 

A crucial aspect of *in vivo* GFB physiology is the crosstalk between GEnC and podocytes. Various studies have highlighted that GEnC and podocyte crosstalk is fundamental for the GFB to function as a filtration barrier [9,10]. For example, the secretion of vascular endothelial growth factor (VEGF) by podocytes affects GEnCs and is an important mechanism to prevent albuminuria [11]. In addition, secretion of the endothelial growth factor Angiopoietin-1 by podocytes stabilizes the glomerular capillaries [12,13]. Furthermore, GEnC have been shown to interact with podocytes via the secretion of exosomes [14]. Moreover, activation of protein kinase C by endothelium-derived thrombomodulin, protects against the development of podocyte injury [15]. In addition to these known examples, more knowledge about the paracrine crosstalk between GEnC and podocytes is likely to lead to a further understanding of *in vivo* GFB (patho)physiology. 

Currently, our understanding of the interaction between GEnC and podocytes is limited due to the small number of studies using *in vitro* models that truly recapitulate the in vivo physiology of the glomerulus [16,17,18,19,20]. Until recently, *in vitro* studies investigating the pathogenic mechanisms of glomerular cell injury used mainly 2D monocultures of e.g., GEnC or podocytes. Organ-on-a-chip technology has been widely used to successfully study elements of the physiology of the heart, lung, liver, and kidney [16,17,18,19,20,21,22,23,24]. Organ-on-a-chip technology has a high potential to create biologically-relevant and complex *in vitro* models of organs and tissues; in our case the glomerulus, [25,26]. In these models, complex glomerular biology can be mimicked by co-culturing GEnC and podocytes in spatial arrangements that recapitulate the physiological tissue architecture. In addition, an important advantage of organ-on-a-chip technology compared to simpler models such as Transwell inserts, is the possibility to eventually implement important conditions like laminar flow and trans-glomerular filter pressure gradients. A recent publication described a glomerulus-on-a-chip model using amniotic fluid-derived podocytes and cultured podocytes and GEnC on top of each other in a single channel [17]. Of note, however, amniotic fluid-derived podocytes are not routinely applicable for high-throughput use. Moreover, this set-up also precludes stimulation or treatment of one specific cell-type, as well as easy experimental separation and subsequent individual analysis of the two cell types. Importantly, whether podocytes and GEnCs affect each other by crosstalk mechanisms was actually not investigated in the previously developed glomerulus-on-a-chip models [16,17,18,19,20]. 

Given the deficits of the systems presented so far, the current study aimed to develop a glomerulus-on-a-chip model that can be used to investigate the effects of crosstalk between GEnC and podocytes on the GFB as a whole, as well as the effects of crosstalk on the phenotype (e.g., gene expression) of the individual glomerular cell types separately. For this purpose, we designed a glomerulus-on-a-chip model with functional filtration capacity by co-culturing conditionally immortalized mouse GEnC and podocyte cell lines on opposite sides of a semi-permeable membrane. The use of conditionally immortalized cell lines in a tailored microfluidic device gives future possibility for high-throughput application. We demonstrate that culturing of podocytes together with GEnC affects gene expression profiles and the morphology and functional phenotype of both GEnC and podocytes, and also improves the filtration barrier of the *in vitro* GFB. 

## 2. Materials and Methods

### 2.1. Microfluidic Chip Design and Assemblance

**Figure 1:**
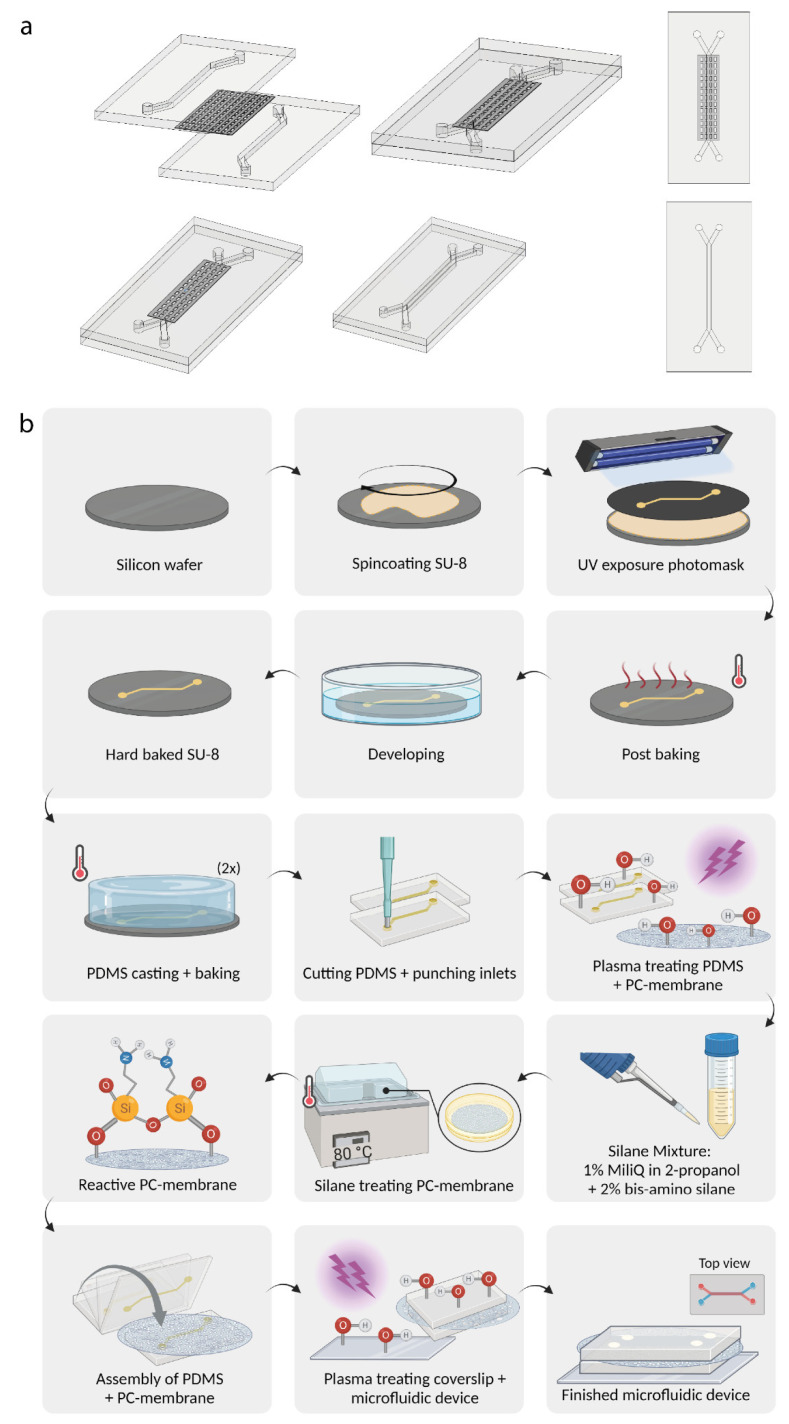
Overview of the production of the glomerulus-on-a-chip device. (**a**) Schematic design of the design of the glomerulus-on-a-chip device. (**b**) Schematic overview of the experimental steps to manufacture the SU-8 molds and polycarbonate membranes in the microfluidic device. PC: polycarbonate. Figure 1b was created using Biorender.com.

Figure 1a. shows a schematic overview of the chip design for the co-culture of GEnC and podocytes. In our chip model, the endothelial and podocyte compartment are separated using a track-etched polycarbonate membrane (8 µm pore size, porosity (void volume) min-max: 4–20%) (WHA155846, Sigma-Aldrich, Zwijndrecht, The Netherlands). The SU-8 master mold was fabricated by patterning a negative photoresist (SU-8 2150, Microchemicals GmbH) on a 4-inch silicon wafer (Si-Mat Silicon Materials, Germany) using conventional photolithography. The resulting SU-8 pattern was about 180 ± 10 µm measured using DektakXT^®^ stylus profilometer (Bruker, Billerica, MA, USA). After fabrication, a silanization process was performed by placing the mold in Trichloro(1H,1H,2H,2H-perfluorooctyl)silane (Sigma-Aldrich, Zwijndrecht, The Netherlands) vapor overnight, leaving the mold ready for PDMS casting. Microfluidic organ-on-a-chip devices were subsequently produced as described previously [27]. In brief, endothelial and podocyte compartments were cast with polydimethylsiloxane (PDMS) (Sylgard 184, Sigma-Aldrich, Zwijndrecht, The Netherlands) in a 10:1 ratio (*w*/*w*) to curing agent. The PDMS mixture was subsequently degassed using a vacuum pump for 10 min and rested for 5 min at room temperature (RT). This step was repeated three times, whereafter the PDMS was cured for 2.5 h at 65 °C. After curing, the PDMS was cut to size and the inlets were punched using a 1.2 mm sized puncher (Harris Uni-Core, Sigma-Aldrich, Zwijndrecht, The Netherlands). 

When assembling the two-layer chips, a modified version of the protocol provided by Sip and Folch was used [28]. The experimental steps to fabricate the SU-8 master mold and coat the polycarbonate membrane are visualized in Figure 1b. In order to create chemical reactivity, the polycarbonate membranes were first oxygen plasma treated using a PDC-32-G-2 plasma cleaner (Harrick Plasma, 1 min, power high). The membranes were subsequently treated using a 2% bis-amino-silane (413,356, Sigma-Aldrich, Zwijndrecht, The Netherlands) solution in isopropanol with 1% H_2_O for 20 min at 80 °C to bind reactive silane groups to the polycarbonate membrane. The membranes were afterwards washed with isopropanol, cured for 30 min at 65 °C and immersed for 30 min in 70% EtOH at RT in small aluminium trays (57 × 16 mm, 40.8 mL) (Avantor, Arnhem, The Netherlands). The endothelial and podocyte PDMS compartments were subsequently oxygen plasma treated to activate the reactive groups of the PDMS (1 min, power high) and directly bonded to the polycarbonate membrane. To ensure an irreversible bond between the reactive silane groups on the polycarbonate membrane and the activated reactive groups of the PDMS compartments, the bond was cured overnight at 65 °C. Finally, the PDMS device was plasma-bonded to a 24 × 50 mm glass cover slide (Avantor, Allentown, PA, USA) (1 min, power high) for 1 h at 65 °C. To create a hydrophilic PDMS surface, the microfluidic device was subsequently oxygen plasma treated (1 min, power high). To ensure a clean cell culturing environment, the microfluidic device and its channels were washed with 70% (*v*/*v*) ethanol. As the podocyte compartment was blocked by the polycarbonate membrane after assembly, the corresponding two inlets were punctured with a sterile p20 pipet tip. After washing, the ethanol was removed and the microfluidic device was washed with sterile PBS. Hereafter, a p20 pipette was used to reach the inlets of the microfluidic channels when handling the microfluidic device. 

### 2.2. Cell Culture 

Conditionally immortalized mouse podocytes (MPC-5) and conditionally immortalized mouse glomerular endothelial cells (mGEnC) were cultured as described previously [29,30]. In brief, MPC-5 and mGEnC were grown at permissive conditions at 33 °C to allow proliferation. To induce differentiation in the microfluidic device, cells were transferred to 37 °C with removal of IFNγ from the growth medium. For the experiments to determine the optimal coating for the cells to adhere and differentiate on the polycarbonate membrane outside the chip, three different coating strategies were tested; (1) 3 h at 37 °C with 1 µg/cm^2^ bovine fibronectin (Thermofisher Scientific, Breda, The Netherlands), (2) 3 h at 37 °C with 1 mg/mL collagen A (i.e., functionally the same as Collagen I) (Merck, Schiphol-Rijk, The Netherlands) or (3) 1 h using 2 mg/mL polydopamine (H8502, Sigma-Aldrich, Zwijndrecht, The Netherlands) in 10 mM Tris-HCl (pH 8.5) at RT, washed with MilliQ, dried for 1 h at 65 °C and subsequently coated for 1 h using 1 mg/mL collagen A at 37 °C. When MPC-5 and mGEnC were seeded in the microfluidic device, the polycarbonate membrane was coated using coating strategy 3, as described above. Two microfluidic devices were stored in a 100 × 20 mm petri dish during culturing (Corning, Sigma-Aldrich, Zwijndrecht, The Netherlands). MPC-5 was seeded into the bottom channel of the chip with a density of 1 × 10^6^ cells/mL. Chips were immediately flipped 180° for 3 h to allow the MPC-5 to adhere to the polycarbonate membrane. After 3 h, chips were flipped back, the medium was placed on top of the microfluidic device to prevent evaporation, and MPC-5 were grown in the device for 14 days to ensure complete differentiation. Every 2 days, the medium was refreshed by adding fresh medium to the channels and fresh medium was placed on top of the microfluidic device. In case of a slanted microfluidic device, which resulted in the inability of the medium to create a surface tension, a custom designed 3D printed lid (Supplementary Figure S2) was inserted in the podocyte compartment inlets. MPC-5 need 14 days to ensure complete differentiation, whereas mGEnC need only 7 days. Therefore, seven days after seeding of MPC-5, mGEnC were seeded with a density of 2 × 10^7^ cells/mL in the top channel of the chip device. Following seeding of mGEnC in the chips, a 1:1 mixture of mGEnC and MPC-5 medium was used to culture the cells. MPC-5 and mGEnC were co-cultured for the remaining 7 days at 37 °C to ensure complete differentiation of both cell types. Every 2 days, the medium was refreshed by adding fresh medium to the channels and fresh medium was replaced on top of the microfluidic device. For the experiments with CellTracker, MPC-5 were stained for 30 min at 37 °C with 12.5 µM CMPTX CellTracker Red and mGEnC with 12.5 µM CMFDA CellTracker Green in serum-free media. 72 h upon seeding of MPC-5, confocal images were acquired as described below.

### 2.3. Immunofluorescent Stainings 

Cells were fixed for 15 min at RT by replacing the medium in the channels with 2% paraformaldehyde (PFA) with 4% sucrose in PBS to stain against von Willebrand Factor (vWF) and synaptopodin. For staining with wheat germ agglutinin (WGA), the cells were fixed for 10 min at RT by replacing the medium with 90% ice-cold acetone. When cells were fixed with PFA, the cells were washed three times with PBS and cells were subsequently permeabilized for 10 min at RT using a 0.3% Triton X-100 solution in 1× PBS. Thereafter, cells were washed three times with PBS and blocked for 30 min at RT by replacing the PBS in the microfluidic channel with blocking solution (2% BSA, 2% FBS and 0.2% fish gelatin in PBS). In case of acetone fixation, cells were washed three times with PBS upon fixation and PBS was replaced by a 1% BSA solution as blocking solution for 30 min at RT. Biotinylated WGA lectin (1:1000 dilution, Vectorlabs, Burlingame, CA, USA) and primary antibodies for vWF (1:25 dilution, DAKO, A0082, rabbit polyclonal) and synaptopodin (1:5 dilution, Progen, 65,194, mouse monoclonal) were diluted in blocking solution and incubated for 1 h at RT by replacing the respective blocking solutions in the microfluidic channels. Streptavidin-conjugated Alexa 594, goat anti-rabbit Alexa 488 and goat anti-mouse IgG1 Alexa 488 (both from Thermofisher Scientific, Breda, The Netherlands) were diluted in blocking solution (1:200) and incubated for 45 min in the dark at RT upon washing the cells three times with PBS. Actin Green 488 or Actin Red 555 (2 drops per 1 mL blocking solution, Thermofisher Scientific, Breda, The Netherlands) were used to stain the actin cytoskeleton and were incubated for 60 min in the dark at RT. Cell nuclei were stained using 2 µg/mL Hoechst 33,342 (Thermofisher Scientific, Breda, The Netherlands) in blocking solution for 5 min at RT. 

### 2.4. Microscopy and Image Analysis

All fluorescent images were acquired using a Zeiss LSM900 inverted confocal laser scanning microscope with Airyscan 2 with a EC Plan-Neofluar 10x/0.45 or a Plan-Apochromat 40x/1.2 water long-distance objective. The surface area of the podocyte foot processes was quantified using the FiloQuant plug-in of FIJI (version 1.53c). In short, a threshold was applied to the images, wherein FiloQuant was used to shave off the protrusions. After, the shaved image was subtracted from the thresholded image and the area of the resulting picture was measured (Supplementary Figure S3). For measuring the total fluorescent intensity of the WGA glycocalyx, Z-stacks were made with a slice interval of 1 µm using a Plan-Apochromat 40x/1.2 water objective. Fluorescent intensity of the WGA Z-stack was measured per slice using FIJI. Three-dimensional reconstructions were made using the 3D viewer plug-in of FIJI. 

### 2.5. Barrier Integrity Assay

To validate the barrier integrity of the chip, 0.5 mg/mL 40-kDA sulphated dextran-FITC and 155-kDA dextran-TRITC (Sigma-Aldrich, Zwijndrecht, The Netherlands) was added to the endothelial compartment and collected from the podocyte compartment after 30 min incubation at 37 °C. Fluorescent intensity was measured at a fluorescent plate reader (Tecan, Infinite Pro2000) and used as a measure of barrier permeability from the endothelial to the podocyte compartment.

### 2.6. RNA Isolation

To collect mGEnC and MPC-5 from the chips, cells were washed once with PBS and subsequently incubated with trypsin for 5 min in the chip. To collect the mGEnC, the top endothelial channel was flushed via the top channel inlet with 1 mL medium, while simultaneously collecting the medium from the top channel outlet. Afterwards, the same procedure was repeated for the bottom channel to collect the MPC-5. Per experimental condition, cells isolated from six chips were pooled into one sample. The following four experimental conditions were tested; (1) mGEnC from monoculture chips, (2) MPC-5 from monoculture chips, (3) mGEnC from co-culture chips, and (4) MPC-5 from co-culture chips. Total RNA isolation was performed using a RNeasy microkit (Qiagen, Hilden, Germany) following the manufacturer’s protocol. RNA concentration and quality were assessed on a Qubit Fluorometer (Thermofischer Scientific, Breda, The Netherlands). 

### 2.7. RNA Sequencing and Analysis

Bulk RNA sequencing was performed using Single Cell Discoveries (Utrecht, The Netherlands). RNA extraction and library preparation followed the CEL-seq2 protocol with a sequencing depth of 20 million reads per sample. R version 3.5.2 was used to analyse the RNA sequencing data. Bulk RNA sequencing count normalization and differential gene expression analysis was performed using the DESeq2 package v3.15 [31]. Significant differentially expressed genes between experimental groups (i.e., co-culture samples vs mono-culture samples, per cell-type) were selected using a log2 fold change threshold of <−1.5 and >1.5. Gene set enrichment analysis (GSEA) was performed using the GSEA v4.1.0 software package [32,33]. Gene functional classification was performed using DAVID tools and functional annotation clustering was performed using GOTERM_BP_DIRECT. Figures were created using BioRender.com.

### 2.8. Statistical Analyses

Data are presented as mean ± SEM. Statistical analysis was conducted with a two-tailed student’s *t*-test when two experimental groups were compared or a one-way ANOVA with Tukey’s Post-Hoc test when three or more experimental conditions were analysed. All statistical analyses were performed using GraphPad Prism version 5.03 (GraphPad Software, Inc., San Diego, CA, USA). A *p*-value of < 0.05 was considered statistically significant.

## 3. Results

### 3.1. Polydopamine and Collagen A Double Coating Is Most Optimal for Glomerular Cell Growth on Polycarbonate Membrane

**Figure 2:**
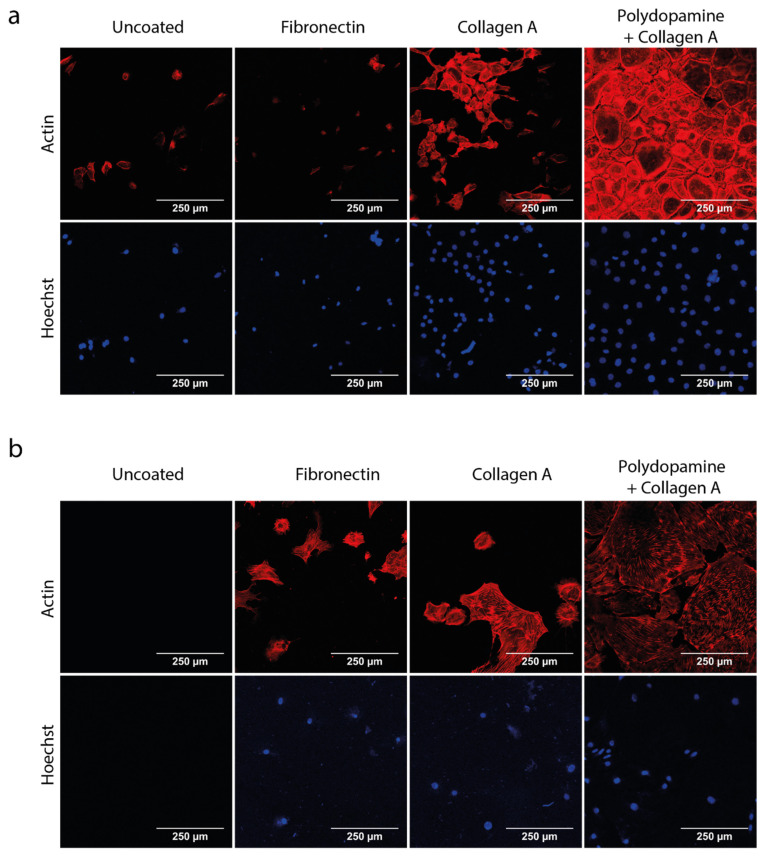
Podocytes and GEnC grow and differentiate on polycarbonate membrane with a polydopamine and collagen A double coating. mGEnC and MPC-5 were seeded on polycarbonate membranes and grown for 1 and 2 weeks, respectively. Membranes were left uncoated, coated with 1 µg/cm^2^ bovine fibronectin, coated with 1 mg/mL collagen A or coated with 2 mg/mL polydopamine and 1 mg/mL collagen A. (**a**) Representative fluorescent images of mGEnC stained for the actin cytoskeleton and Hoechst 33,342 as nuclear staining. (**b**) Representative fluorescent images of MPC-5 stained for the actin cytoskeleton and Hoechst 33,342 as nuclear staining (n = 3).

First, we optimized the coating which could allow for the growth of mGEnC and MPC-5 on the polycarbonate membrane outside the chip. Notably, over the last decade, we have gained extensive experience in 2D monoculture with these two cell lines and exactly know how these cells behave morphologically and functionally [29,34,35]. The following four experimental conditions were investigated: (1) uncoated membranes, (2) membranes coated with fibronectin, (3) membranes coated with collagen A and (4) membranes coated with both polydopamine and collagen A. We found that coating the membrane first with polydopamine and then with collagen A was the optimal membrane coating, as this combination resulted in complete monolayer growth and differentiation for both cell types after their respective differentiation period (i.e., 7 days for mGEnC and 14 days for MPC-5) (Figure 2a,b). In comparison, neither mGEnC nor MPC-5 adhered properly on uncoated membranes. On membranes solely coated with fibronectin, only a small number of MPC-5 were able to grow, whereas mGEnC were completely unable to grow. On membranes coated with collagen A, the growth of mGEnC was improved profoundly but was still insufficient to obtain a complete monolayer of mGEnC. Collagen A coating of the membrane also improved the growth of MPC-5 compared to the fibronectin-coated membrane. Coating of the membranes with both polydopamine and collagen A yielded complete monolayers for both mGEnC and MPC-5. Moreover, MPC-5 displayed a large cell surface area and expressed synaptopodin (data not shown), thereby indicating their complete differentiation [36]. Therefore, the double coating of polycarbonate membranes with polydopamine and collagen A was selected to be used in this study to culture mGEnC and MPC-5 in the glomerulus-on-a-chip device. 

### 3.2. Development of a Co-Culture in the Microfluidic Device

After identifying the optimal membrane coating for culturing mGEnC and MPC-5 on the polycarbonate membrane outside the chip, we investigated the behavior of mGEnC and MPC-5 when co-cultured in our glomerulus-on-a-chip device. We found that mGEnC were seeded into the top channel of the chip, whereas MPC-5 were seeded into the bottom channel of the chip to grow on the bottom side of the membrane. 

**Figure 3:**
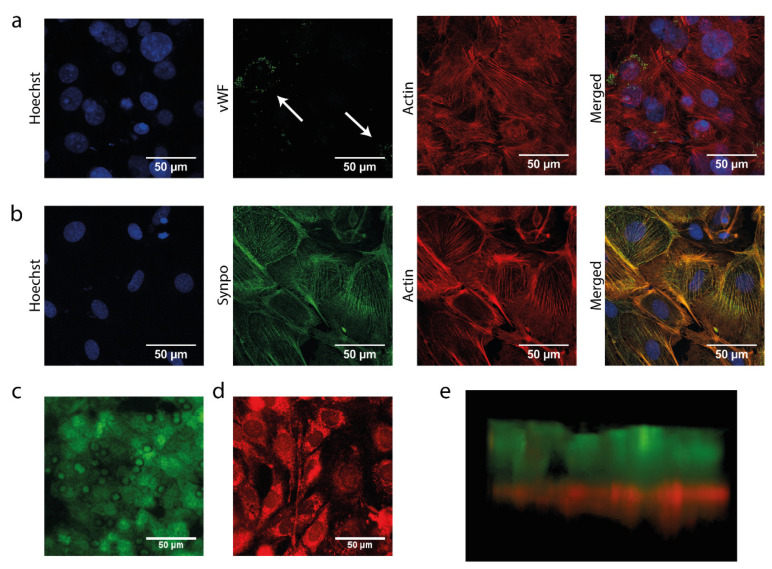
Podocytes and GEnC grow and differentiate in a glomerulus-on-a-chip device. mGEnC were seeded into the top channel of the chip and were allowed to grow for 1 week on the membrane. MPC-5 were seeded in the bottom channel of the chip and were allowed to grow for 2 weeks on the other side of the membrane. (**a**) Representative fluorescent images of mGEnC grown for 1 week in the chips stained for Hoechst 33,342 (blue), vWF (green), and actin (red). (**b**) Representative fluorescent images of MPC-5 grown for 2 weeks in the chips stained for Hoechst 33342 (blue), synaptopodin (green), and actin (red). (**c**) Representative fluorescent image of mGEnC stained with CellTracker Green 72 h after seeding. (**d**) Representative fluorescent image MPC-5 stained with CellTracker Red 72 h after seeding. (**e**) 3D reconstruction of the Z-stack made of the mGEnC-MPC-5 co-culture in the chip 72 h after seeding. Prior to seeding, mGEnC were stained with CellTracker Green and MPC-5 with CellTracker Red.

We confirmed that mGEnC in monoculture also formed a monolayer on the polydopamine and collagen A double coated membrane in the chip (Figure 3a). Furthermore, mGEnC displayed high levels of vWF expression in Weibel-Palade bodies, which confirmed the complete differentiation of mGEnC in the chip (Figure 3a, second panel from the left, indicated by the arrows) [29]. MPC-5 also formed a complete monolayer when cultured in monoculture in the chip on the bottom side of the membrane coated with both polydopamine and collagen A (Figure 3b). Moreover, MPC-5 showed high expression levels of the podocyte-maturity marker synaptopodin, which validated the correct differentiation of the podocytes in our chip device [36]. Upon verifying the separate growth and differentiation of mGEnC and MPC-5 in our chip device, we also confirmed that mGEnC and MPC-5 simultaneously formed intact monolayers on opposite sides of the membrane in our chip device using fluorescent CellTracker dyes (Figure 3c–e). These findings validated the successful co-culture of mGEnC and MPC-5 in our glomerulus-on-a-chip model. 

### 3.3. Co-Culture Affects Podocyte Morphology and Increases Glycocalyx Thickness

**Figure 4:**
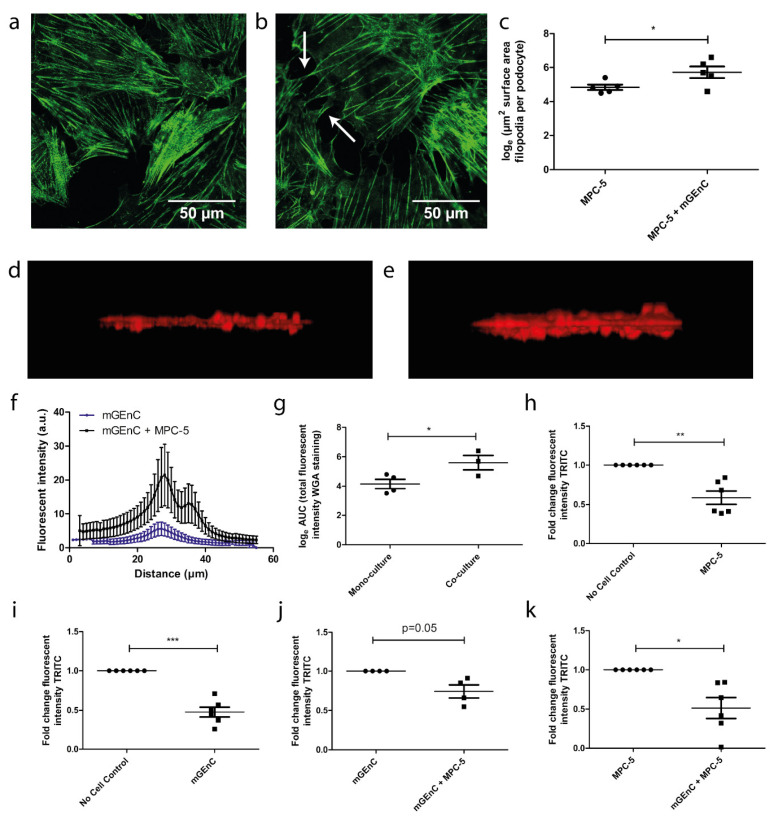
Co-culture of GEnC and podocytes affect podocyte morphology, glycocalyx thickness and barrier function. mGEnC were seeded in the top channel of the chip and were allowed to grow for 1 week on the top of the membrane. One week prior to seeding mGEnC, MPC-5 were seeded in the bottom channel of the chip and were allowed to grow for 2 weeks on the bottom side of the membrane. (**a**) Representative fluorescent image of synaptopodin staining in MPC-5 in monoculture. (**b**) Representative fluorescent image of synaptopodin staining in MPC-5 in co-culture. (**c**) Quantification of filopodia surface area MPC-5 in mono- and co-culture. (**d**) 3D reconstruction of the WGA staining of mGEnC cultured alone. (**e**) 3D reconstruction of the WGA staining of mGEnC cultured together with MPC-5. (**f**) Quantification of the fluorescent intensity of the WGA staining per Z-stack slice. (**g**) Calculation of the AUC of the fluorescent intensity of the WGA staining. (**h**) Fold change in fluorescent intensity of 155-kDA dextran-TRITC in chips without cells and chips with a monoculture of mGEnC. (**i**) Fold change in fluorescent intensity of 155-kDA dextran-TRITC in chips without cells and chips with a monoculture of MPC-5. (**j**) Fold change in fluorescent intensity of 155-kDA dextran-TRITC in chips with a monoculture of mGEnC and chips with a co-culture of mGEnC and MPC-5. (**k**) Fold change in fluorescent intensity of 155-kDA dextran-TRITC in chips with a monoculture of MPC-5 and a co-culture of mGEnC and MPC-5. * *p* < 0.05, ** *p* < 0.01, *** *p* < 0.001.

After ascertaining that mGEnC and MPC-5 could be co-cultured in our chip device and showed a differentiated endothelial and podocyte phenotype, we investigated the effect of co-culture on the morphology and functional phenotype of mGEnC and MPC-5. First, we observed an altered podocyte morphology in co-culture, which was characterized by extended podocyte protrusions and an increased number of filopodia (Figure 4a,b). Moreover, the surface area of podocyte filopodia was increased when cultured together with mGEnC (Figure 4c, *p* < 0.05). We subsequently used the lectin WGA to study the effect of co-culture on the formation of the endothelial glycocalyx in our chip model. WGA detects both hyaluronic acid (HA) and unsulfated domains within heparan sulfate (HS). HA is the main structural component of the glycocalyx and responsible for maintaining the gel-like structure of the glycocalyx. In addition, HS is the main functional component of the glycocalyx and prevents for example the binding of leukocytes and cytokines to the healthy endothelium, whereas it facilitates binding of leukocytes and cytokines under inflammatory conditions [2]. Notably, the formation of the endothelial glycocalyx increased when mGEnC were co-cultured with MPC-5 compared to mGEnC cultured in monoculture (Figure 4d,e). In addition, the intensity of the WGA staining was significantly increased when mGEnC were cultured together with MPC-5 compared to mGEnC cultured in monoculture, suggesting a denser glycocalyx in co-culture (Figure 4f,g, *p* < 0.05). 

### 3.4. Co-Culture of GEnC and Podocytes Improves Functional Barrier Integrity of the Glomerulus-on-a-Chip Model

Maintaining the integrity of the charge and permselective barrier is an important characteristic for the correct functioning of the glomerulus *in vivo*, and glomerular injury is associated with increased GFB permeability and eventually albuminuria and proteinuria. Thus, after investigating the effect of co-culture on the morphology of the individual cell types, we investigated the effect of co-culture on the functional integrity of the filtration barrier in our chip model. To determine the functional barrier integrity, we measured the passage rate of 155-kDA dextran-TRITC from the top to the bottom channel of the microfluidic device, i.e., from endothelial channel to podocyte channel. The monoculture of mGEnC and MPC-5 resulted in a ~50% decreased passage of 155-kDA dextran-TRITC across the barrier compared to chips without cells (Figure 4h,i, *p* < 0.001 and *p* < 0.01, respectively). Importantly, the passage rate of 155-kDA dextran-TRITC across the barrier was further decreased in chips with a co-culture of mGEnC and MPC-5 compared to chips with a monoculture of either mGEnC or MPC-5 (Figure 4j,k, *p* = 0.05 and *p* < 0.05). We obtained similar findings using the negatively charged 40-kDA sulphated dextran-FITC dye (Supplementary Figure S1). These findings highlight the importance of both podocytes and GEnC as a functional part of the filtration barrier in our chip model. 

### 3.5. Co-Culture of GEnC and Podocytes Regulate Pathways Involved in Cell Differentiation and Cell Adhesion

**Figure 5:**
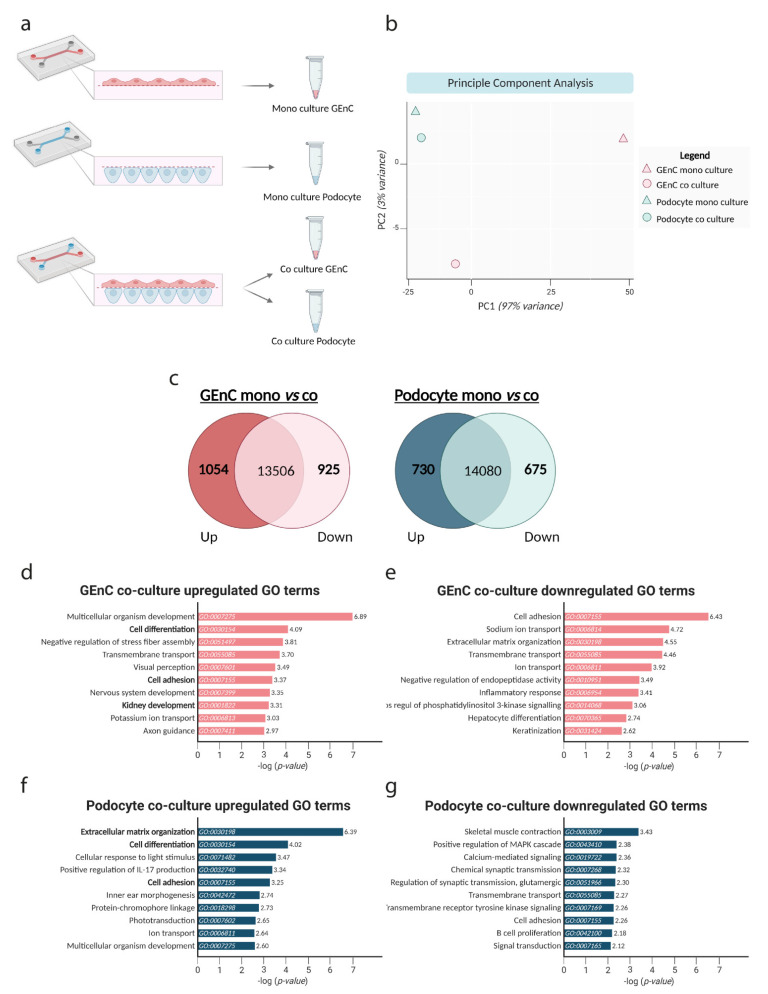
Co-culture of GEnC and podocytes activates pathways involved in cell differentiation and cell adhesion. (**a**) Schematic overview of experimental design. mGEnC and MPC-5 were cultured in chips alone, or the cells were cultured in co-culture whereafter the mGEnCs and MPC-5 were separately isolated. (**b**) Principle component analysis (PCA), visualizing the variance in gene expression profiles between the different conditions. (**c**) Venn-diagram showing the total number of genes and significantly up- and down-regulated genes in mGEnC (red) and MPC-5 (blue). Genes were selected using a logFC of <−1.5 and >1.5. (**d**) The top 10 upregulated GO terms in biological processes in mGEnC and (**e**) the top 10 downregulated GO terms in biological processes in mGEnC as a result of co-culture. (**f**) The top 10 upregulated GO terms in biological processes in MPC-5 (**g**) and the top 10 downregulated GO terms in biological processes in MPC-5 as a result of co-culture. Gene functional annotation was obtained via DAVID tools and the top 10 most upregulated and top 10 most downregulated GO terms are shown based on the calculated -log *p*-values.

Finally, we studied the effect of co-culture in the glomerulus-on-a-chip device on gene expression of both GEnC and podocytes by performing whole transcriptome bulk RNA-seq analysis. The experimental setup is visualized in Figure 5a. Principal component analysis (PCA) revealed that co-culture profoundly affected the RNA expression profile of both MPC-5 and mGEnC (Figure 5b). Upon selecting genes with a log fold change in gene expression of <−1.5 or >1.5, we observed that 1054 on a total of the 15,485 sequenced genes were significantly upregulated in mGEnC and 925 genes out of the 15,485 sequenced genes were significantly downregulated when cultured together with MPC-5 (Figure 5c). In addition, 730 out of the 15,485 sequenced genes were significantly upregulated and 675 out of the 15,485 sequenced genes were significantly downregulated in MPC-5 in co-culture with mGEnC compared to monoculture. We performed functional gene classification to investigate which biological processes appeared regulated in MPC-5 and mGEnC as a result of co-culture. Notably, pathways involved in cellular differentiation, cell adhesion and kidney development were regulated in mGEnC as a result of co-culture with MPC-5 (Figure 5d,f). In addition, pathways involved in extracellular matrix organization, cell differentiation and cell adhesion were affected in MPC-5 when cultured together with mGEnC (Figure 5e,g). Taken together, co-culture of GEnC and podocytes in our novel glomerulus-on-a-chip system profoundly affected the transcriptome of both GEnCs as well as podocytes.

## 4. Discussion 

In this study, we report the design and application of a novel microfluidic glomerulus-on-a-chip model, designed to enable studying cross-talk between cell types. In this glomerulus-on-a-chip model glomerular endothelial cells and podocytes were cultured on different sides of a semi-permeable membrane in separate channels in a microfluidic chip design, thereby reconstituting the *in vivo* glomerular architecture. We were able for the first time to successfully reconstitute the filtration barrier function of the GFB in a microfluidic device by culturing two conditionally immortalized glomerular cell lines. In order to mirror the *in vivo* situation, we chose to use a very thin poly-carbonate membrane as physical support to mimic the glomerular basement membrane and establish the glomerular filtration barrier. The microfluidic setup was optimized using a poly-carbonate membrane, ensuring proper chemical reactivity of the three layers (PDMS-membrane-PDMS) to induce bonding, leakage-free membrane integration and practical assembly steps (Figure 1). We showed that co-culture of differentiated GEnCs and podocytes in this device alters both their morphology and transcriptome, the thickness of the glycocalyx as well as cross-membrane barrier integrity, in comparison to monocultures. 

Crosstalk between GEnC and podocytes is known to be crucial to maintain the integrity of the GFB [9,10]. However, studies using advanced *in vitro* models to accurately study these mechanisms are still scarce. The GFB is often considered as an unchanging biological entity. However, over the last decade, an increasing number of studies have suggested that the dynamic interaction between the different components of the GFB plays a crucial role in glomerular health and glomerular disease [13,37,38]. By developing a microfluidic glomerulus-on-a-chip model, we can demonstrate the importance of co-culture of GEnC and podocytes on the morphology and functional phenotype of both cell types. In addition, we showed that co-culture resulted in a thicker glycocalyx. The glycocalyx is a key structural element of the GFB and plays a role in immune cell adhesion, growth factor binding, glomerular structural integrity, specific endothelial function as well as overall glomerular barrier function [2,3,4]. Interestingly, although structurally different from endothelial cells, podocytes also produce a glycocalyx, the thickness of which is. affected by diabetic conditions, although the contribution of podocyte glycocalyx to the charge-dependent barrier function of GFB remains elusive [3,39,40]. Generally, a decreased thickness of the endothelial glycocalyx is associated with albuminuria and has been previously observed in the pathogenesis of kidney pathology such as diabetic nephropathy and chronic kidney disease [41,42]. The findings presented in this study reveal that co-culture of glomerular endothelial cells and podocytes, and thus probably glomerular crosstalk, is important to maintain a thicker glycocalyx and subsequently to prevent the development of albuminuria during renal health and disease.

In addition to the effect of glomerular crosstalk on the glycocalyx, our experiments have also revealed the effect of co-culture on cellular differentiation in general. Our findings following bulk RNA sequencing experiments provide a further insight, that co-culture led to profound alterations of biological pathways in both cell types. Moreover, the effect on cellular differentiation was further substantiated by differences in the surface area of podocyte filopodia. Of note, the retraction and shrinkage of podocyte foot processes, known as podocyte foot process effacement, is a pathological characteristic observed in a wide variety of glomerular diseases [43,44]. In addition, podocyte foot process effacement is often interpreted as evidence that podocyte injury is the initial step in the pathogenesis of glomerular diseases. However, the data shown in this study might suggest that podocyte foot process effacement could also be the result of pathogenetic mechanisms originating in the glomerular endothelium instead.

Clearly, the aim of using organ-on-a-chip technology in this study was not to recapitulate a completely functional kidney outside the human body. It was rather a strategy to recapitulate a crucial and complex substructure of the kidney, namely the filtration barrier, which cannot be addressed with conventional 2D monoculture strategies or cannot be readily investigated in animal models. In the current study, we primarily wanted to demonstrate that the interaction between GEnC and podocytes could be studied in our custom glomerulus-on-a-chip device, and that we could measure differences in the clinically relevant outcome parameter like filtration barrier integrity. Whereas podocytes form the architectural backbone and provide mechanical stability to the barrier, glomerular endothelial cell functions are regulated by podocytes with respect to e.g., growth and differentiation via paracrine factors such as VEGF [45,46]. This perfectly illustrates the interplay between physical and functional properties of podocytes and GEnC in the GFB. Additional features and improvements may be added to the current chip model in future studies depending on the research question, e.g., addition of a channel containing mesangial cells. 

Over the past few years, different glomerulus-on-a-chip models have been developed via distinct methodologies [16,17,18,19,20]. For example, one study used a commercially available organ-on-a-chip platform [17], while other studies used soft lithography to develop a custom-made glomerulus-on-a-chip model [16,18,19,20]. Whether podocytes and GEnCs separated by a membrane affect each other by crosstalk mechanisms was not investigated in the previously developed glomerulus-on-a-chip models. In the current study, we decided to use soft lithography to design our glomerulus-on-a-chip model, as this allowed us to tailor the design of the microfluidic device to answer our research question: the effect of co-culture on the phenotype of GEnC and podocytes. Designing a glomerulus-on-a-chip model consisting of two parallel microfluidic channels, separated by a polycarbonate membrane, allowed us to study the two cell types separately and also enabled the easy separation of the two cell types for RNA-seq analysis after co-culture. Whereas some studies cultured isolated glomeruli in their microfluidic device, other studies cultured stem cell-derived podocytes or amniotic fluid-derived podocytes. In the current study, we were able for the first time to successfully reconstitute the filtration barrier function of the GFB in a microfluidic device by culturing two conditionally immortalized glomerular cell lines. We chose these two cell lines because we already had comprehensive knowledge about both cell lines and therefore knew precisely how they should grow and differentiate [29,34,35]. Importantly, demonstrating that conditionally immortalized glomerular cell lines can be used to construct a glomerulus-on-a-chip opens the possibility for a high through-put application, which is challenging when culturing amniotic fluid-derived podocytes or isolated glomeruli.

In conclusion, the glomerulus-on-a-chip model we developed can be used in future studies to further investigate the crosstalk between GEnC and podocytes *in vitro*. An improved understanding of cellular interactions in the glomerulus will greatly advance our understanding of the molecular mechanisms in GFB health and disease, and might eventually lead to the discovery of new therapeutic strategies for the treatment of glomerulopathies. In addition, the model can be used to perform disease-modelling and subsequent drug screening studies to reduce the use of animal studies in drug development. For example, plasma from patients with primary FSGS or membranous nephropathy (MN) could be added to our glomerulus-on-a-chip model to recapitulate the pathogenesis of FSGS and MN [47]. Furthermore, the podocyte injury inducing compounds Adriamycin or puromycin aminonucleoside (PAN) could be used in future studies to mimic the pathophysiology of FSGS [48,49]. Moreover, an alternative strategy to use the glomerulus-on-a-chip model for disease modelling would be the culture of GEnC and podocytes derived from stem cells from patients, e.g., those with Alport syndrome or other inherited glomerulopathies, even enabling personalized approaches [50]. Finally, the glomerulus-on-a-chip model developed in this study can also be used to study the molecular mechanisms underlying the migration of immune cells through the GFB by introducing immune cells to the endothelial compartment. As glomerular infiltration of immune cells is an important pathogenic mechanism of immune-related glomerular diseases, the model developed in the current study might provide new insights in the pathogenesis of e.g., diabetic nephropathy and glomerulonephritis [51,52].

## Data Availability

The data presented in this study are available on request from the corresponding author.

## Supplementary Materials

The following supporting information can be downloaded at: https://www.mdpi.com/article/10.3390/bios13030339/s1, Figure S1: Co-culture of mGEnC and MPC-5 is required for barrier function against 40-kDa sulphated dextran-FITC. mGEnC were seeded in the top channel of the chip and were allowed to grow for 1 week on the top of the membrane. One week prior to seeding mGEnC. MPC-5 were seeded in the bottom channel of the chip and were allowed to grow for 2 weeks on the bottom side of the membrane. Fold change in fluorescent intensity of 40-kDA sulphated dextran-FITC in chips without cells, in chips with a mono-culture mGEnC, in chips with a mono-culture of MPC-5 and chips with a co-culture of mGEnC of MPC-5. ** *p* < 0.01; Figure S2: Schematic model of custom designed 3D printed lids; Figure S3: Additional figures corresponding Figure 4a–c. Podocytes are visualized either when cultured in mono culture (top row) or in co-culture with mGEnC (down row).
