# Supplementary material for: Co-Culture of Glomerular Endothelial Cells and Podocytes in a Custom-Designed Glomerulus-on-a-Chip Model Improves the Filtration Barrier Integrity and Affects the Glomerular Cell Phenotype
Source: Biosensors (Basel). 2023 Mar 3;13(3):339. doi: 10.3390/bios13030339 (PMC10046631; doi:10.3390/bios13030339)
Supplement: Supplementary file 1 [file biosensors-13-00339-s001.zip › biosensors-2128316-supplementary.pdf]

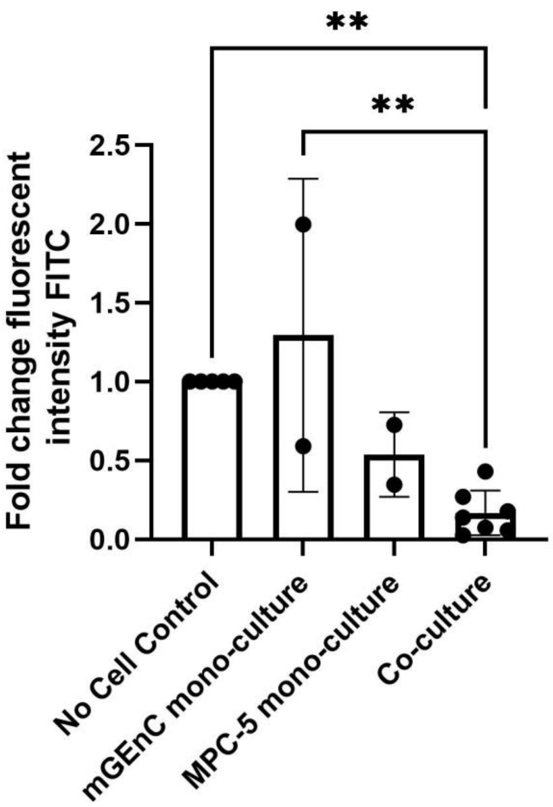

Figure S1. Co-culture of mGEnC and MPC-5 is required for barrier function against 40-kDa sulphated dextran-FITC. mGEnC were seeded in the top channel of the chip and were allowed to grow for 1 week on the top of the membrane. One week prior to seeding mGEnC. MPC-5 were seeded in the bottom channel of the chip and were allowed to grow for 2 weeks on the bottom side of the membrane. Fold change in fluorescent intensity of 40-kDA sulphated dextran-FITC in chips without cells, in chips with a mono-culture mGEnC, in chips with a mono-culture of MPC-5 and chips with a co-culture of mGEnC of MPC-5. ** $p < 0.01$.

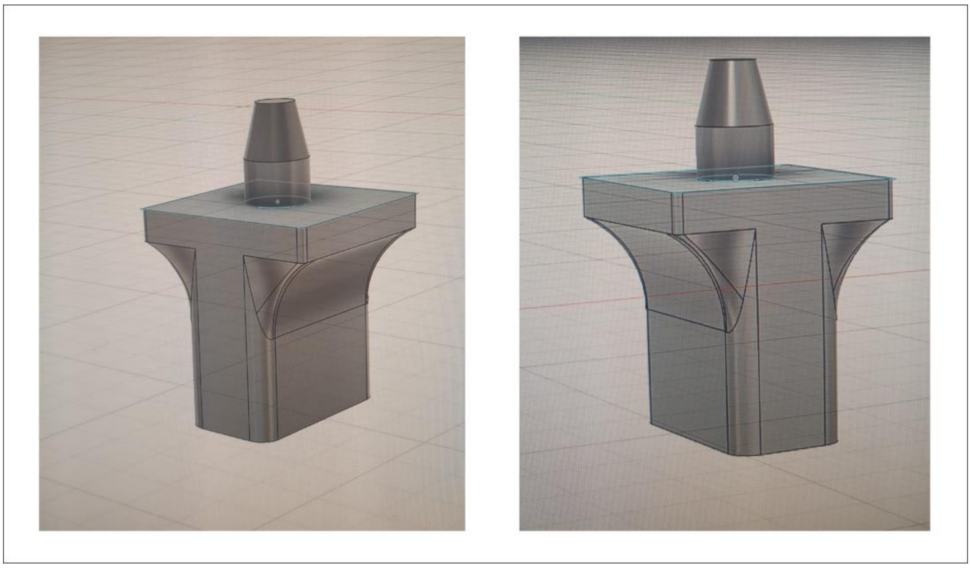

Figure S2. Schematic model of custom designed 3D printed lids.

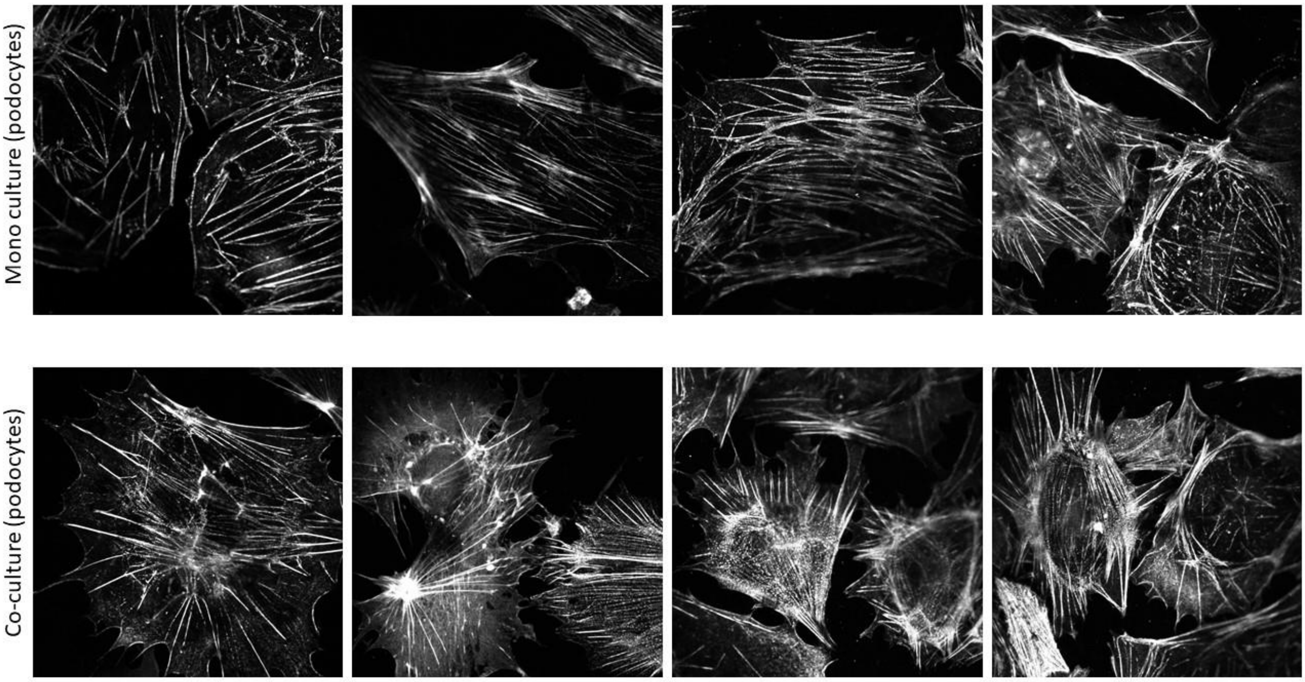

Figure S3. Additional figures corresponding Fig.4a+b+c. Podocytes are visualized either when cultured in mono culture (top row) or in co-culture with mGEnC (down row).
